# Supplementary material for: Feasibility of Developing Audiovisual Material for Training Needs in a Vietnam Orphanage: A Mixed-Method Design
Source: Int J Environ Res Public Health. 2023 Feb 10;20(4):3118. doi: 10.3390/ijerph20043118 (PMC9966681; doi:10.3390/ijerph20043118)
Supplement: Supplementary file 1 [file ijerph-20-03118-s001.zip › ijerph-2038093-supplementary.pdf]

Document S1: Ad hoc survey

First of all, thank you very much for your collaboration in this project! The objective of this questionnaire is to know your opinion about the viability and applicability of the audiovisual material that we have made to be used in the orphanages of Vietnam. This questionnaire is part of a study carried out within the subject of Final Degree Project at the Universidad San Jorge de Zaragoza.

CONTENT OF THE VIDEOS

The content shown (topics addressed) in the videos is appropiate and can contribute to meeting the real needs in the orphanage:
1= totally disagree
2 = disagree
3 = neither agree nor disagree
4 = agree
5 = totally agree

The content of the videos is understandable / comprehensible for the physiotherapists of the orphanage:
1= totally disagree
2 = disagree
3 = neither agree nor disagree
4 = agree
5 = totally agree

The content of the videos (especially those related to feeding, transfers and clothing) is understandable / comprehensible to the caregivers in the orphanage:
1= totally disagree
2 = disagree
3 = neither agree nor disagree
4 = agree
5 = totally agree

The content of the videos and the proposals made in them are applicable in the orphanage:
1= totally disagree
2 = disagree
3 = neither agree nor disagree
4 = agree
5 = totally agree

The use of these videos can contribute to improve the training and assistance that physiotherapists offer to children in the orphanage:
1= totally disagree
2 = disagree
3 = neither agree nor disagree
4 = agree
5 = totally agree

FORMAT OF THE VIDEOS
The length of each video seems to be adequate:
1= totally disagree
2 = disagree
3 = neither agree nor disagree
4 = agree
5 = totally agree

The materials used in the videos are adequate and could be used in the orphanage in Vietnam:
1= totally disagree
2 = disagree
3 = neither agree nor disagree
4 = agree
5 = totally agree

The written language used in the videos is adequate and contributes to a better understanding of the content:
1= totally disagree
2 = disagree
3 = neither agree nor disagree
4 = agree
5 = totally agree

OPEN-ENDED QUESTIONS
- What did you think of the videos? Which aspects do you consider the most positive?
- Would you change something? Which aspects do you think that could be improved?
- Personal opinion + comments or suggestions you would like to make.
